# HIV-1 infection, response to treatment and establishment of viral latency in a *novel* humanized T cell-only mouse (TOM) model

**DOI:** 10.1186/1742-4690-10-121

**Published:** 2013-10-24

**Authors:** Jenna B Honeycutt, Angela Wahl, Nancie Archin, Shailesh Choudhary, David Margolis, J Victor Garcia

**Affiliations:** 1 Division of Infectious Diseases, Center for AIDS Research, University of North Carolina School of Medicine, 120 Mason Farm Rd., CB 7042, Genetic Medicine Building 2044, Chapel Hill, NC, USA

## Abstract

**Background:**

The major targets of HIV infection in humans are CD4^+^ T cells. CD4^+^ T cell depletion is a hallmark of AIDS. Previously, the SCID-hu thy/liv model was used to study the effect of HIV on thymopoeisis *in vivo*. However, these mice did not develop high levels of peripheral T cell reconstitution and required invasive surgery for infection and analysis. Here, we describe a novel variant of this model in which thy/liv implantation results in systemic reconstitution with human T cells in the absence of any other human hematopoietic lineages.

**Results:**

NOD/SCID-hu thy/liv and NSG-hu thy/liv mice were created by implanting human fetal thymus and liver tissues under the kidney capsule of either NOD/SCID or NSG mice. In contrast to NOD/SCID-hu thy/liv mice that show little or no human cells in peripheral blood or tissues, substantial systemic human reconstitution occurs in NSG-hu thy/liv. These mice are exclusively reconstituted with human T cells (i.e. T-cell only mice or TOM). Despite substantial levels of human T cells no signs of graft-versus-host disease (GVHD) were noted in these mice over a period of 14 months. TOM are readily infected after parenteral exposure to HIV-1. HIV replication is sustained in peripheral blood at high levels and results in modest reduction of CD4^+^ T cells. HIV-1 replication in TOM responds to daily administration of combination antiretroviral therapy (ART) resulting in strong suppression of virus replication as determined by undetectable viral load in plasma. Latently HIV infected resting CD4^+^ T cells can be isolated from suppressed mice that can be induced to express HIV *ex-vivo* upon activation demonstrating the establishment of latency *in vivo*.

**Conclusions:**

NSG-hu thy/liv mice are systemically reconstituted with human T cells. No other human lymphoid lineages are present in these mice (i.e. monocytes/macrophages, B cells and DC are all absent). These T cell only mice do not develop GVHD, are susceptible to HIV-1 infection and can efficiently maintain virus replication. HIV infected TOM undergoing ART harbor latently infected, resting CD4^+^ T cells.

## Background

SCID-hu thy/liv mice develop a bona-fide human thymic organ and have a marginal level of systemic reconstitution with human T cells [1,2]. The human thymic organoid present in the SCID-hu model is susceptible to HIV infection [3]. However, infection of these animals requires this tissue to be surgically exposed and virus administration via direct injection [4]. HIV injection results in infection of the human thymocytes present but there is no viremia in these mice, thus analysis of virus replication and its effect on thymocytes requires surgical removal of a piece of tissue [4]. Subsequent monitoring of infection over time also requires additional surgical collection of tissue for analysis. Although the use of this model is extremely labor intensive and requires large numbers of animals to make meaningful observations, the SCID thy/liv model has been extensively used to evaluate HIV pathogenesis of the thymus, the effect of HIV on thymocyte development, the establishment of HIV latency in thymocytes *in vivo*, the efficacy of antiviral drugs on thymocytes and the role of auxiliary genes of HIV in virus replication and CD4^+^ thymocyte destruction [5-8].

Following the development of the SCID thy/liv model, several other novel strains of mice have been derived with a higher degree of immune suppression. These include the NOD/SCID and the NOD/SCID common gamma chain null (NSG) strains of immunodeficient mice [1]. Both of these strains have been extensively and successfully used in the derivation of a variety of humanized mouse models [9]. However, neither of these two strains has been extensively used to produce humanized thy/liv implanted mice [10].

Resting CD4^+^ T cells represent a well-characterized reservoir for latent HIV-1 infection, and this reservoir persists long-term despite treatment with highly active antiretroviral therapy (HAART) [11-13]. Incubating resting CD4^+^ T cells with CCL19, secreted by mature dendritic cells, *ex vivo* increases HIV-1 integration efficiency [14]. Additionally, the chemokines CXCL9 and CXCL10, secreted by monocyte-derived cells and induced by IFN-γ production, seem to mediate similar effects in resting T cells [11,14-16]. Secretion of IL-7 by dendritic cells may be important for the survival of memory T cells, and secretion of IL-15 by macrophages and other mononuclear phagocytes is important for the low level of proliferation necessary to maintain a resting memory pool over time [17]. Thus while it is known that several myeloid-derived cell types secrete cytokines and chemokines that facilitate the development of latency and maintain the resting CD4^+^ T cell pool, whether or not these cells are necessary for the establishment of latency *in vivo* remains unknown [18].

With the long-term goal of obtaining a better understanding of HIV replication, CD4^+^ T cell depletion, HIV latency and persistence *in vivo*, we sought to study HIV-1 in a humanized mouse model that possesses human T cells but is devoid of human myeloid (and B) cells. To this effect, we implanted human thymus and liver into NOD/SCID and NSG mice. In this study we show that whereas NOD/SCID hu thy/liv mice do not develop high levels of systemic reconstitution with human cells, NSG hu thy/liv mice develop high levels of human T cells in the peripheral blood. Remarkably, flow cytometric analysis of blood and tissues demonstrate the complete absence of human B and myeloid cells in these mice. Interestingly, in contrast to mice reconstituted with human peripheral blood mononuclear cells (PBMC) and some other types of humanized mice [19,20], these T cell-only mice (or TOM) do not develop signs of GVHD. In addition, TOM are readily susceptible to HIV infection after parenteral exposure and can sustain high levels of HIV replication. Virus replication can be efficiently suppressed by antiretroviral therapy and HIV latency is established in resting T cells.

## Results and discussion

**Figure 1:**
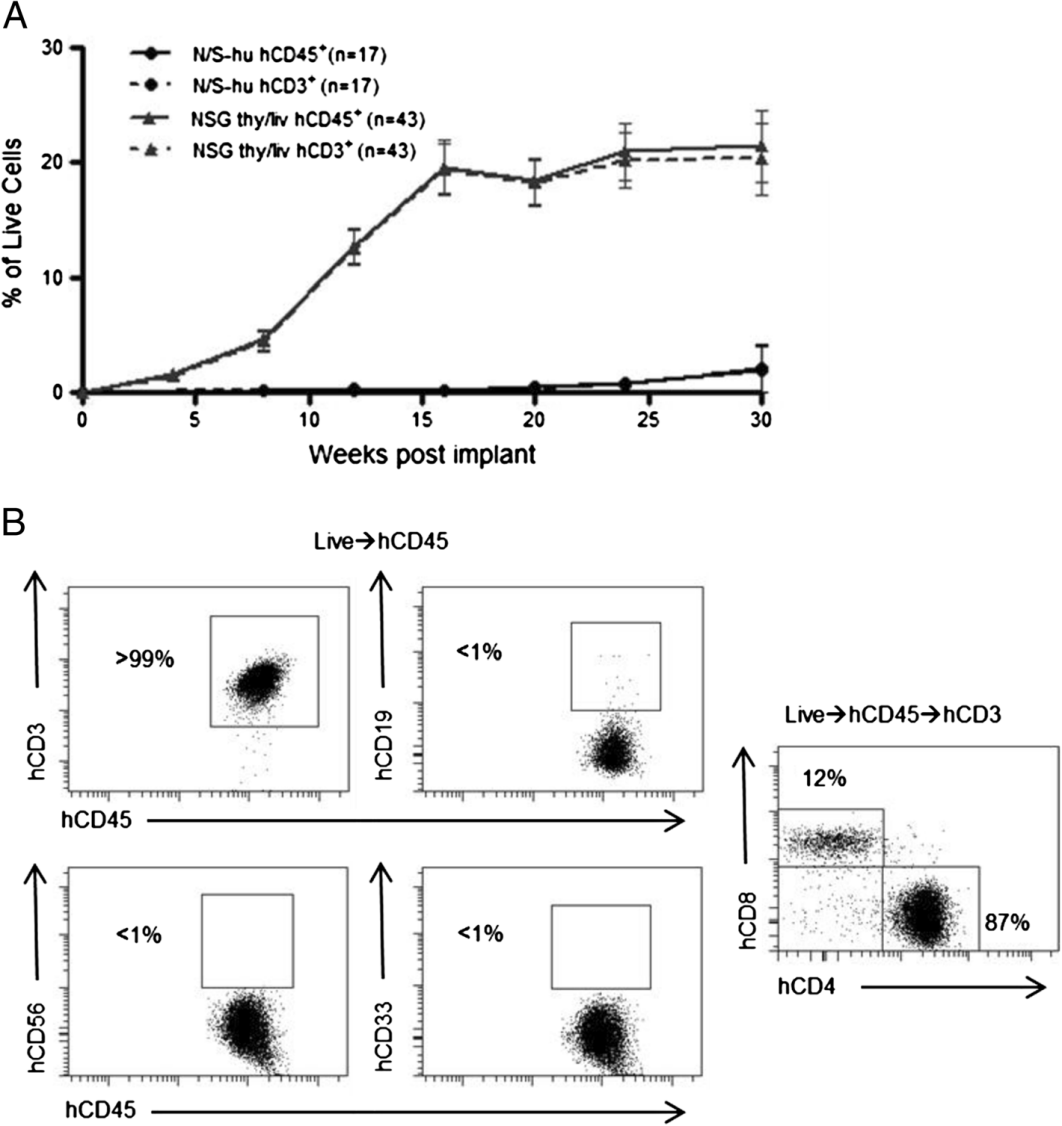
**Analysis of the peripheral blood (PB) of thy/liv implanted NSG mice demonstrates long-term reconstitution with human T cells. A)** Flow cytometric analysis of the PB of NOD/SCID-hu (N/S-hu) (black circles) and NSG thy/liv (gray triangles) mice demonstrates systemic reconstitution of implanted NSG mice with human cells (CD45^+^; solid line) and human T cells (CD3^+^; dashed line). Gating strategy: live cells→human CD45→human CD3. **B)** Flow cytometric analysis of cells from PB of a representative NSG-hu thy/liv mouse (29 weeks post-implant) demonstrates these mice are exclusively reconstituted with human T cells with greater than 99% of cells expressing human CD3. There is a lack of B cells (CD19^+^), natural killer cells (CD56^+^), and myeloid cells (CD33^+^) in the PB of TOM. PB CD3 expressing cells were then analyzed for CD4 and CD8 expression levels. The majority of CD3^+^ cells in PB expressed CD4 (87%).

SCID-hu (thy/liv) mice have been extensively used as an *in vivo* model to study HIV infection of the thymus [7]. Since the original development of the SCID-hu thy/liv model, new and improved strains of immunodeficient mice like NOD/SCID and NSG have been developed [6,8]. We implanted human thymus and liver into NOD/SCID and NSG mice to determine whether or not these strains would be an improvement over the SCID-hu model. We then monitored the peripheral blood (PB) of these mice over time by polychromatic flow cytometry for the presence of human cells (hCD45). While the NOD/SCID implanted mice, like the original SCID-hu mice, did not have significant levels of human cells in their PB, the implanted NSG mice had substantial levels of human reconstitution as determined by presence of human CD45 in their PB (Figure 1A). Furthermore, human cells present in the PB of these mice were identified as T cells by their cell surface expression of human CD3 (Figure 1B). Interestingly, exhaustive analysis for the presence of other lymphoid or myeloid human cells did not reveal any significant levels of these cells in the PB of any animals analyzed. Specifically, we did not detect human B cells (CD19^+^), human natural killer cells (CD56^+^), or human myeloid cells (CD33^+^) in the peripheral blood of NSG-implanted mice (Figure 1B). Additionally, there were no human dendritic cells present in these mice (Lin^-^/HLA-DR^hi^, data not shown). Thy/liv implanted NSG mice showed sustained production of human T cells that reached approximately 20% in peripheral blood for up to 30 weeks (the last time point analyzed). Over this period, no signs of graft-versus-host disease (GVHD) were observed. Additionally, some animals were followed for up to 12 months post-implant (the last time point analyzed). These animals were found to sustain 20-30% human T cells in the PB even at these late time points (n = 2, data not shown). From these results, we concluded that implantation of human thymus and liver into NSG mice results in sustained and exclusive production of human T cells *in vivo*.

**Figure 2:**
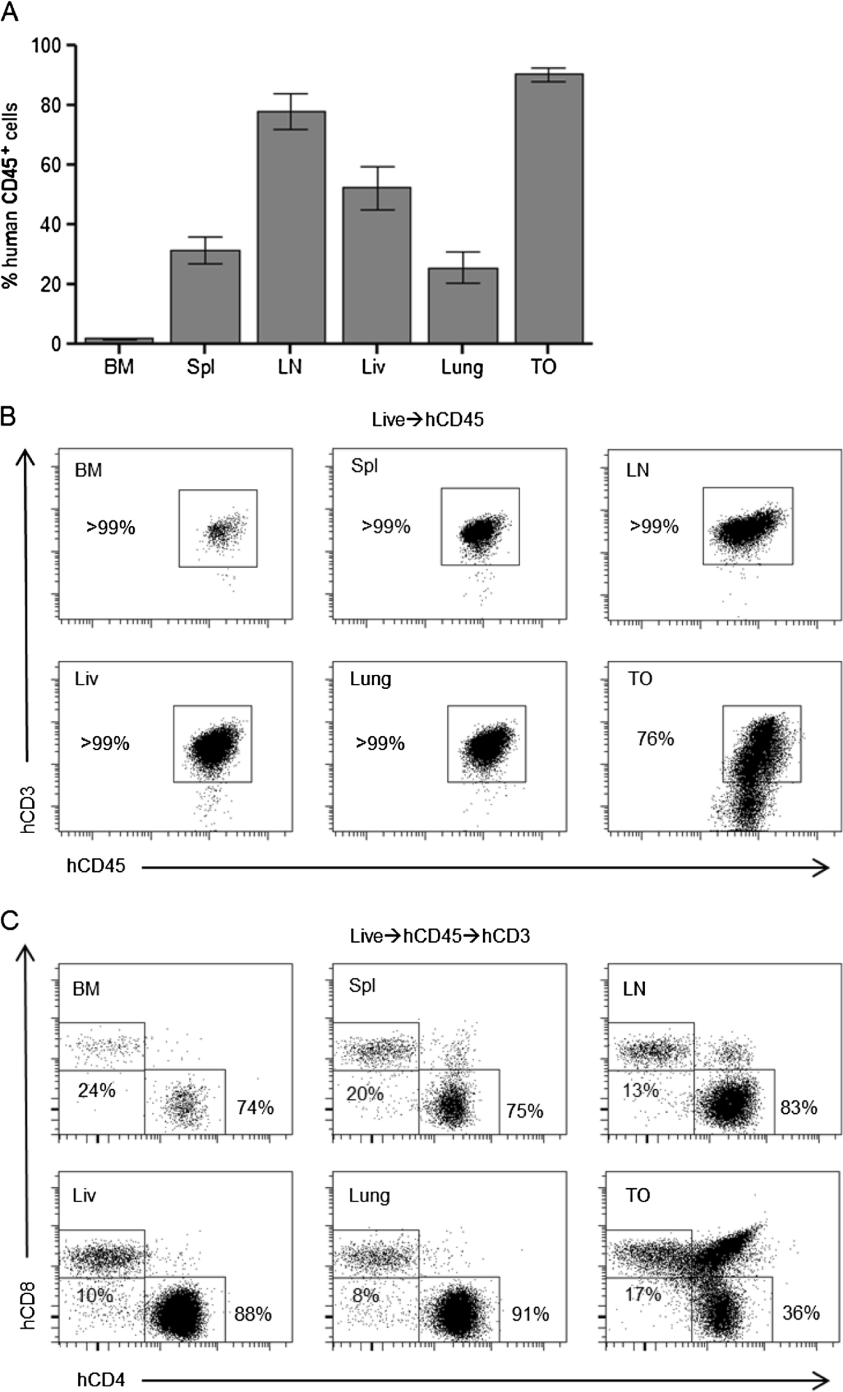
**Peripheral tissues of implanted thy/liv NSG mice are extensively reconstituted with human T cells. A)** Flow cytometric analysis of cells harvested from the bone marrow (BM), spleen (Spl), lymph nodes (LN), liver (Liv), lung, and the thymic organoid (TO) of implanted thy/liv NSG mice (n = 15) demonstrated reconstitution with human cells (CD45^+^). **B)** Flow cytometric analysis of tissues harvested from an implanted thy/liv NSG mouse (29 weeks post-implant) demonstrated that all organs were reconstituted exclusively with human T cells or thymocytes. **C)** Flow cytometric analysis of tissues and PB harvested from an implanted thy/liv NSG mouse demonstrated that all organs were reconstituted with both CD4^+^ and CD8^+^ T cells.

In SCID-hu mice, human cells are almost exclusively found in the thymic organoid, with little reconstitution of PB or tissues [21]. To determine the systemic distribution of the human cells present in NSG mice, mononuclear cells were isolated from the bone marrow, spleen, lymph nodes, liver, lung, and the thymic organoid. Flow cytometric analyses demonstrated that the spleen, lymph nodes, liver, lung, and thymic organoid were robustly reconstituted with human cells (CD45^+^) (Figure 2A). Consistent with the lack of hematopoietic stem cell engraftment in these mice, low levels of human cells were observed in the bone marrow. To determine if the tissues were repopulated with human T cells, we used flow cytometry to assess the presence of hCD3. We observed that greater than 99% of human cells in the bone marrow, spleen, lymph nodes, liver, and lung were human T cells (CD3^+^) (Figure 2B). All tissues and the peripheral blood were reconstituted with both CD4^+^ and CD8^+^ T cells (Figure 2C). In the thymic organoid, we also observed a preponderance of human CD3^+^ cells at various stages of differentiation [22] (Figure 2B). We also noted an abundance of double positive (CD4^+^/CD8^+^) T cells, consistent with normal thymopoiesis (Figure 2C). Additionally, the spleen, small and large intestines of TOM were analyzed by flow cytometry and immunohistochemistry. In the spleen, T cells were distributed throughout the entire organ in small groups of cells similar to those observed in BLT humanized mice [23]. In contrast to all other tissues analyzed (including the spleen) we found no significant levels of human cells present in the gastrointestinal tract of these animals (data not shown).

**Figure 3:**
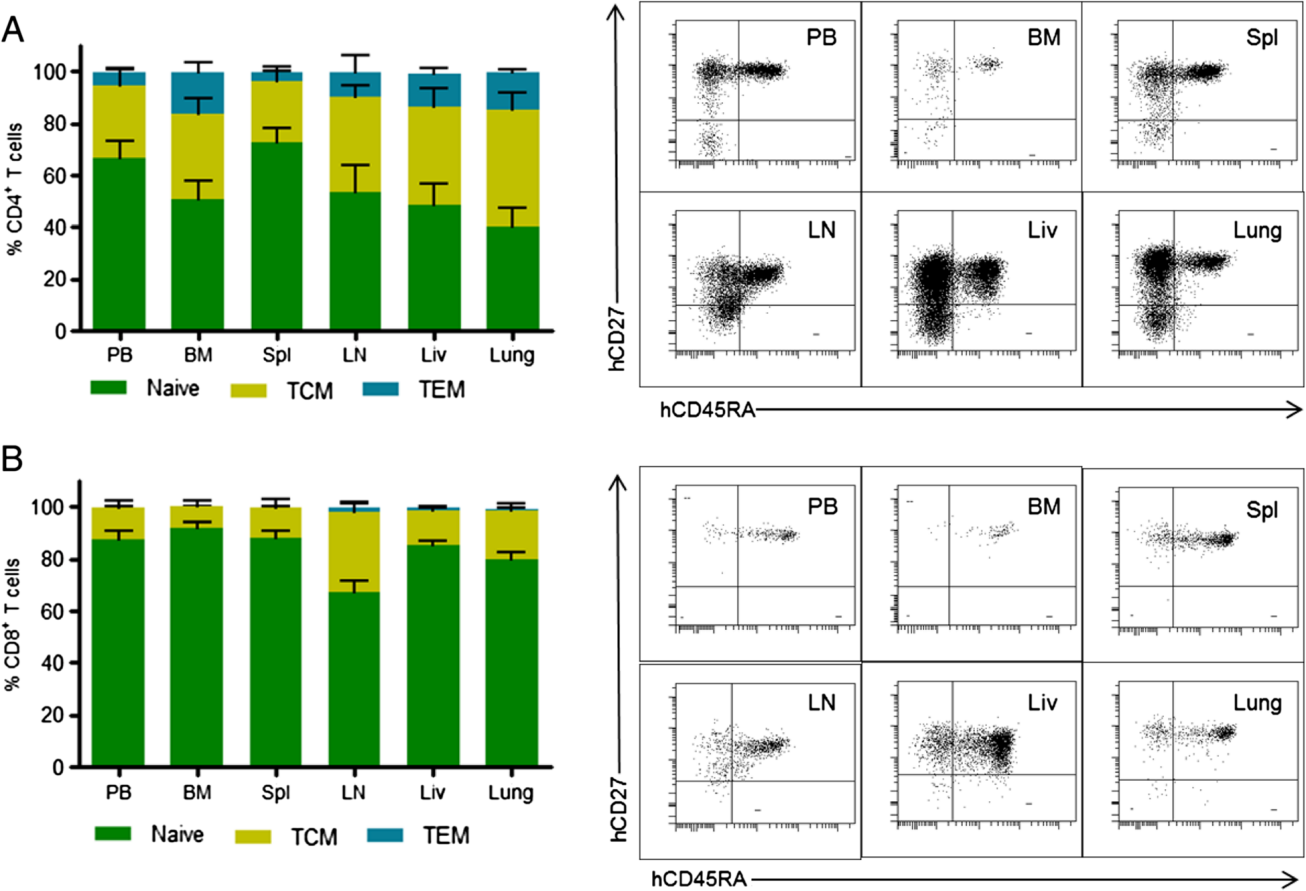
**Naïve/memory phenotype of T cells in the PB and tissues of TOM.** (left) The distribution of central memory, naïve and effector memory phenotypes of CD4^+^**(A)** and CD8^+^**(B)** T cells in the PB and tissues of TOM (n = 7) was determined with flow cytometry. Animals were approximately 35 weeks post-implantation on average at time of harvest. (right) Naïve T cells were defined as CD45RA^+^CD27^+^, central memory T cells (TCM) were defined as CD45RA^neg^CD27^+^, and effector memory T cells (TEM) were defined as CD45RA^neg^CD27^neg^. Error bars represent the Mean + SEM.

We further investigated the phenotypes of the human T cells present in PB and tissues. Both CD4^+^ and CD8^+^ T cells from PB and tissues of these mice had a predominantly naïve phenotype, expressing both human CD45RA and CD27 (Figure 3). Additionally, we found CD4^+^ T cells with central memory (CD45RA^-^/CD27^+^) and effector memory (CD45RA^-^/CD27^-^) phenotypes (Figure 3A). Within the CD8^+^ T cell population, mainly naïve and central memory phenotypes were observed, with effector memory phenotypes being extremely rare in this population (Figure 3B). These results demonstrate that the human CD4^+^ T cells in these mice have a normal developmental phenotype.

**Figure 4:**
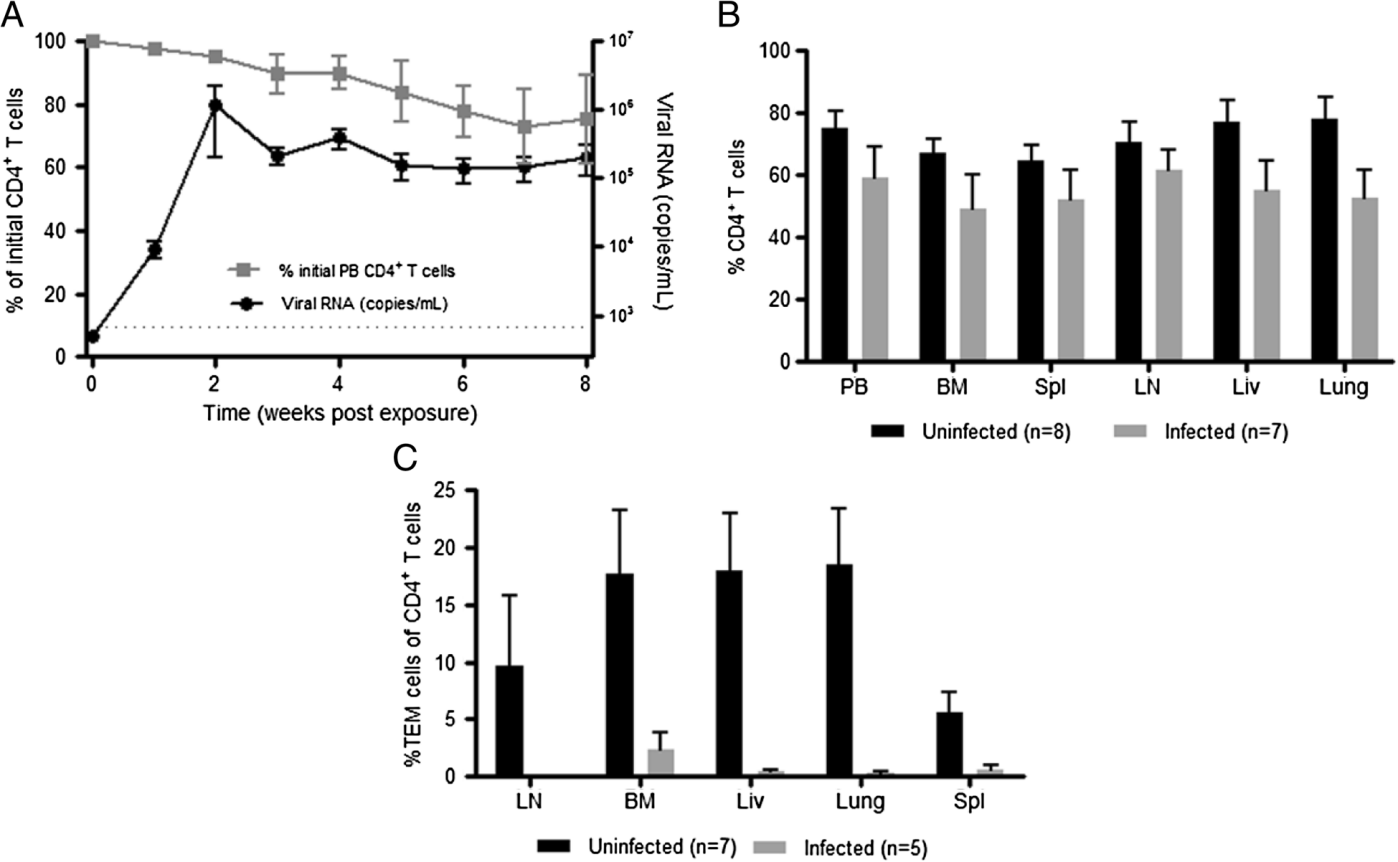
**HIV-1 replication and CD4**^**+ **^**T cell depletion in TOM. A)** TOM (n = 8) were parenterally exposed to HIV-1 and the viral load monitored in PB plasma (black circles). Changes in CD4^+^ T cell levels in PB were measured over time using flow cytometric analysis (gray squares). Gating scheme: live cells → hCD45 → hCD3 → CD4. The limit of detection for viral load is indicated with a dashed line. **B)** The percentage of CD4^+^ T cells present in the tissues of infected (gray bars) and non-infected (black bars) TOM were analyzed using flow cytometric analysis. Gating scheme: live cells → hCD45 → hCD3 → CD4. **C)** HIV infection results in a reduction in the levels of effector memory cells within the CD4^+^ T cell population of infected mice (n = 5, grey bars) versus uninfected (n = 7, black bars) mice. Infected animals were approximately 48 weeks post-implantation at the time of harvest. For all graphs, error bars represent Mean ± SEM.

Once we established the systemic reconstitution of TOM, we tested whether or not they could support HIV-1 replication. Eight TOM were infected with a single dose of cell-free HIV-1_JR-CSF_, a CCR5-tropic isolate administered parenterally. We then monitored the plasma of TOM for the presence of HIV-1 RNA as previously described [13,24]. HIV-RNA was detected in the plasma of TOM one week after exposure and high levels of viremia were maintained over time (Figure 4A). We also monitored CD4^+^ T cell depletion in PB, a measure of HIV pathogenesis. The presence of HIV-RNA in PB correlated with a subsequent drop in circulating CD4^+^ T cells (Figure 4A). Similar decreases in CD4^+^ T cell levels were observed in all tissues analyzed consistent with systemic virus spread (Fig. 4B). Phenotypic analysis of the remaining CD4^+^ T cells in the infected mice demonstrated the specific depletion of effector memory T cells (TEM) cells in PB and tissues (Figure 4C). These results demonstrate the susceptibility of TOM to HIV infection, their ability to sustain high levels of virus replication and the depletion of CD4^+^ TEM cells from PB and tissues of infected animals.

**Figure 5:**
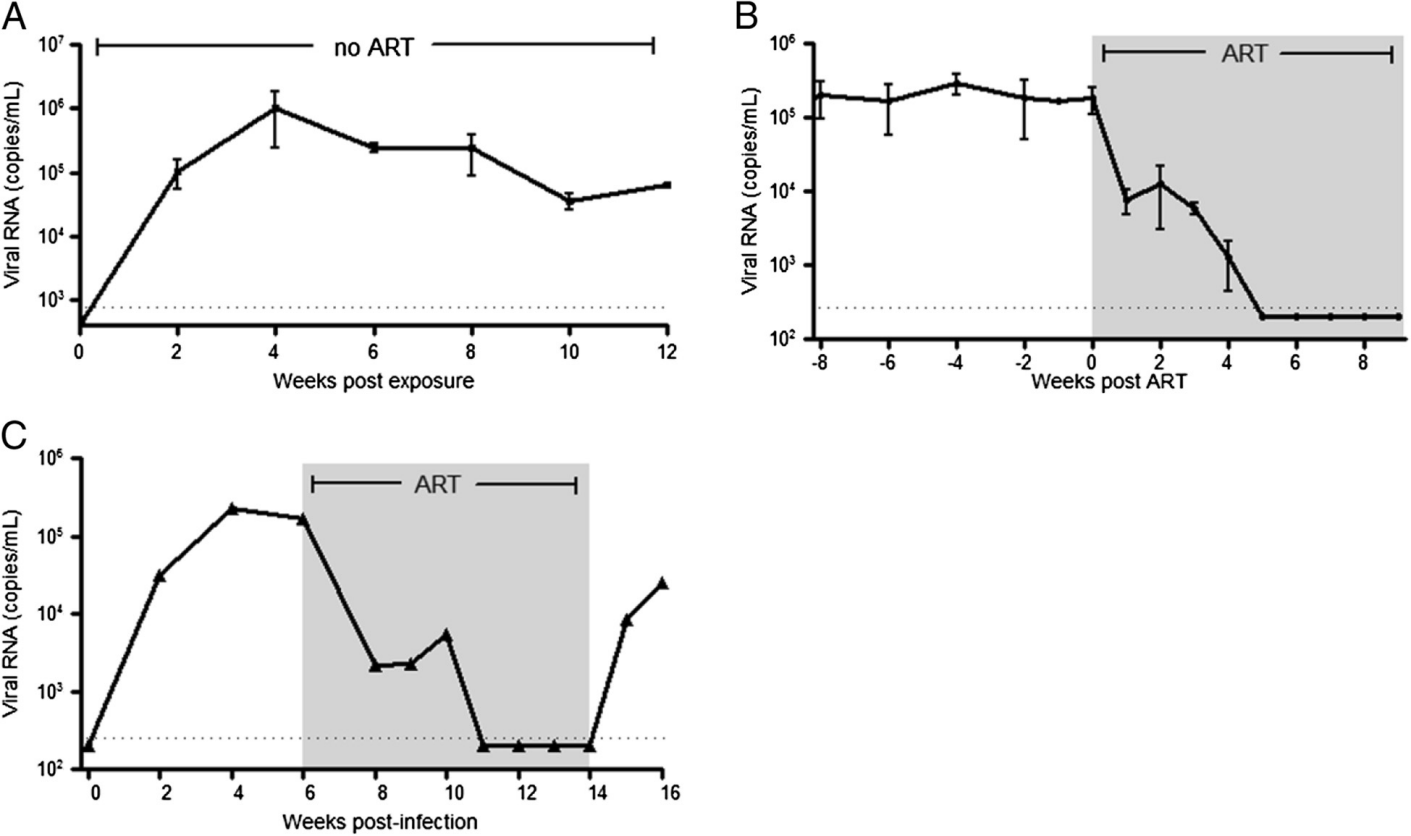
**There is sustained HIV replication in TOM that can be efficiently suppressed by ART. A)** Sustained levels of plasma HIV-RNA in infected TOM (n = 5). **B)** The plasma viral load of infected TOM (n = 4) pre- and post-initiation of ART treatment demonstrated the ability of ART to suppress viremia in these mice. ART consisted of daily injections of TDF, FTC and Raltegravir. **C)** Plasma viral load from an infected TOM dropped below the limit of detection and remained undetectable for the duration of ART. As seen in humans, viremia rebounded following treatment interruption. The limit of detection of the assay is indicated with a dashed line. Error bars in both graphs represent the Mean ± SEM.

Having established the capacity of TOM to support HIV infection, we proceeded to determine if virus replication could be suppressed by combination antiretroviral therapy (ART). For this purpose, HIV infected TOM were treated daily with a combination of FTC, TDF and raltegravir. This regimen has been shown by our laboratory to effectively suppress viral replication in the humanized BLT mouse model [13]. To assess the effectiveness of ART in infected TOM , levels of HIV-RNA were monitored in the plasma throughout treatment. ART administration resulted in a rapid reduction in the levels of plasma HIV-RNA in all treated animals (Figure 5B). Five weeks after initiation of treatment the levels of HIV-RNA in plasma were below the detection limit of our assay (750 RNA copies/ml). These results demonstrate the efficacy of ART in controlling HIV replication in humanized TOM. However, even long-term ART does not result in virus eradication and treatment interruption in patients leads to viral rebound [25]. To determine if this also occurs in TOM, we executed an analytical therapy interruption in one HIV infected and ART suppressed mouse. One week after treatment cessation, viral RNA was detected in the plasma of this mouse demonstrating the expected virus rebound (Figure 5C).

**Figure 6:**
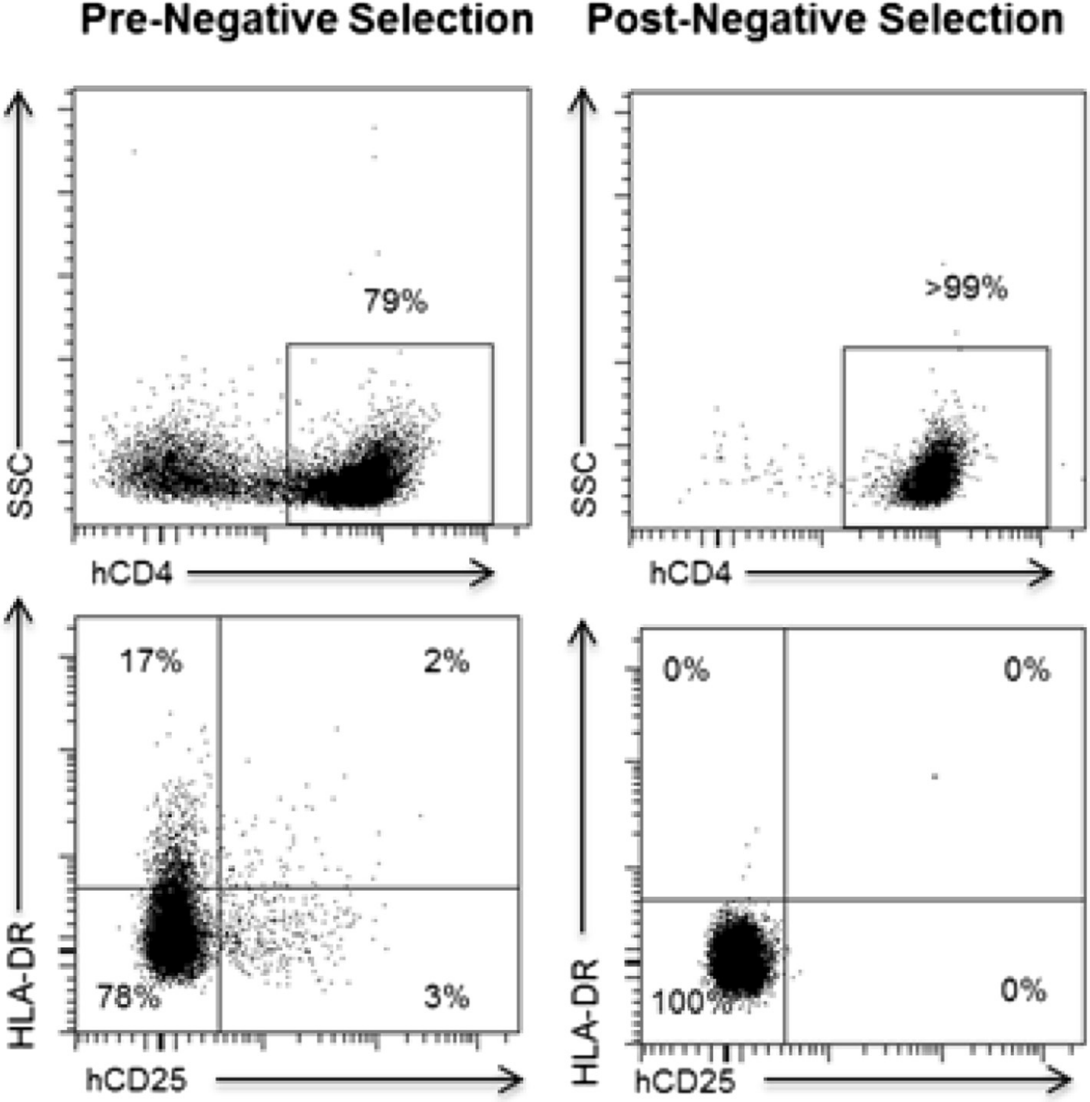
**Resting human CD4**^**+ **^**T cell isolation from TOM.** (Top left) Flow cytometric analysis of cells pooled from the different tissues of a TOM prior to magnetic negative selection showed the presence of both CD4^+^ and CD4^neg^ cells. (Bottom left) Prior to negative selection CD4^+^ T cells expressed various levels of CD25 and HLA-DR. (Top right) After isolation 99% of the cells obtained were CD4^+^. (Bottom right) Consistent with a resting phenotype, isolated cells were CD3^+^CD4^+^ and did not express CD25 or HLA-DR. Gating strategy: (Top) live→hCD45^+^→hCD3^+^. (Bottom) live→hCD45^+^→hCD3^+^→CD4^+^.

**Figure 7:**
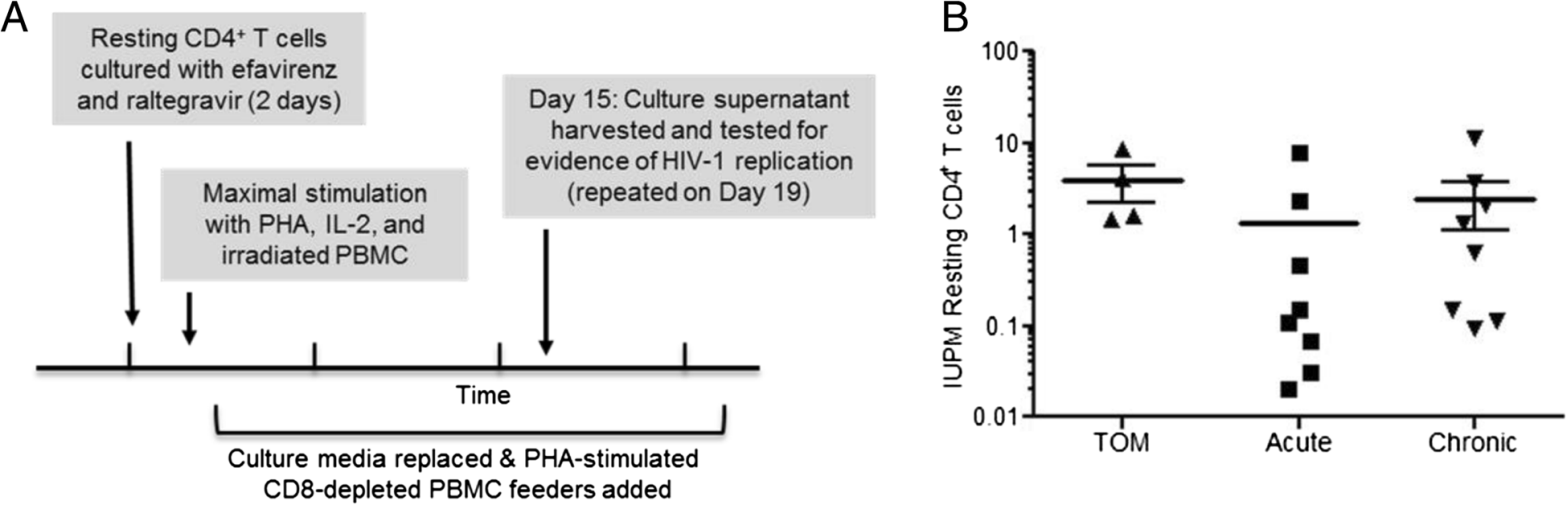
**Latent HIV infection of human resting CD4**^**+ **^**T cells in TOM and human PB.** The frequency of latently infected resting CD4^+^ T cells was measured in resting CD4^+^ T cells isolated from ART-suppressed TOM and PB of suppressed patients that initiated treatment during acute or chronic phases of HIV-1 infection via co-culture of resting cells. **A)** Diagram depicting the resting cell co-culture assay to detect p24 expression after maximal stimulation with PHA. **B)** Comparison of the number of resting HIV infected cells between humanized mice and humans during the acute or chronic phase of infection. The number of infected resting cells in the individual mice was estimated using a maximum likelihood method and values reported in infectious units per million resting cells (IUPM).

In humans, ART results in virus strong virus suppression, increases in CD4^+^ T cell levels and other significant health benefits to patients [26]. Despite this strong virus suppression seen in ART treated patients, HIV persists in resting CD4^+^ T cells that form a long lasting latent reservoir [27]. To determine if HIV could establish a latent reservoir in TOM, we first confirmed the presence of resting human T cells in TOM. To assess the presence of resting human T cells in TOM, we collected cells from PB, bone marrow, spleen, lymph nodes, liver, lung, and thymic organoid from HIV^+^ ART suppressed mice. Cells from all the tissues obtained from each individual mouse were pooled together and human CD4^+^ T cells in each pool of cells corresponding to one individual mouse were analyzed for expression of hCD25 and HLA-DR. This analysis demonstrated the presence of significant numbers of resting human CD4^+^ T cells in TOM (Figure 6). To determine if resting cells were latently infected with HIV, pooled cells from all tissues of each individual ART suppressed mouse were used to isolate resting CD4^+^ T cells by negative selection using magnetic beads [12,13]. After magnetic selection, a highly purified population of resting CD4^+^ T cells, shown by a lack of CD25 and HLA-DR expression was obtained from each individual mouse (Figure 6). To quantify the frequency of latently infected resting CD4^+^ T cells in these mice, we used a protocol validated for the same purpose for use in humans [28]. Specifically, the pooled resting cells from each individual mouse were cultured with efavirenz and raltegravir for two days to prevent any de-novo infection from unintegrated virus that may be present. Next, the resting cells were maximally stimulated in limiting dilutions with PHA, IL-2 and irradiated PBMC from an uninfected donor, followed by co-culture with allogeneic PHA-activated CD8^+^-T-cell-depleted feeder cells. Fifteen days later, cultures were tested for the presence of HIV (Figure 7A). The number and density of cultures was then used in a maximum likelihood method to estimate the number of infectious units per million cells (IUPM). Latently infected cells were obtained from all four animals analyzed (Figure 7B). IUPM values from mice were compared with values obtained from outgrowth assays using resting CD4^+^ T cells of HIV infected patients treated during the acute or chronic phase of infection. The levels of latently infected cells in these mice are within those observed in the chronic patients receiving suppressive ART [29,30]. To confirm that this indeed is induction from latency, as an added control, prior to stimulation, supernatant from resting cell cultures were assayed for P24 and were all found to be negative (data not shown) suggesting that virus recovered from outgrowth experiments originated from latent provirus. These results demonstrate the establishment of HIV latency in TOM and demonstrate that *in vivo* human T cells alone are sufficient for establishing latency.

Although SCID-hu thy/liv animals have been used extensively to study thymopoiesis and HIV-1 infection of the thymus, additional applications of this model has been limited by the lack of peripheral access to the human cells [31,32]. Specifically, in this model a lack of systemic reconstitution with human cells requires invasive surgery for infection and monitoring of virus replication [4]. In one report, low levels of human cells in PB, spleen and lymph nodes of SCID-hu thy/liv implanted mice were noted [33]. However, this required implantation of twenty pieces of human thy/liv tissue under both kidney capsules of each mouse. Using this more invasive implantation strategy combined with 20X more tissue, HIV-1 infection was achieved after IP or intra-implant injection. Using the original implantation strategy described for SCID-hu mice, the use of more immunodeficient mouse strains, like the NSG strain, has overcome the limited systemic reconstitution previously seen in SCID-hu mice. Interestingly, thy/liv implantation of NOD/SCID mice did not result in systemic reconstitution with T cells suggesting that the additional immunosuppression due to the lack of a functional common gamma chain observed in NSG mice resulting in a complete lack of natural killer cells [34] is likely contributing to the increased T cell levels in these mice.

TOM were systemically reconstituted with human T cells. This reconstitution is consistent with the continued production of human T cells from the implanted thy/liv organoid as it showed a substantial and robust population of CD3^+^/CD4^+^/CD8^+^ thymocytes for as long as the animals were examined (1.2 years). Consistent with the lack of cryptopatches in NSG mice [35] TOM showed essentially no significant accumulation of human T cells in the intestinal tract (data not shown). TOM show phenotypically normal CD4^+^ T cell development. However, we noted somewhat limited CD8^+^ T cell development in TOM, with few effector memory CD8+ cells. These differences in the formation of effector phenotypes in the CD4^+^ and CD8^+^ T cell populations may be due to the absence of cytokine signals from professional APCs as well as CD4^+^ helper T cells that limit CD8^+^ T cell activation/differentiation [22]. The reduction in the percentage of CD4^+^ T cells with an effector memory phenotype pre- and post- HIV infection cannot be attributed to differences in the source of donor tissues since tissue from a total of 11 different donors were used to generate the mice used for these experiments.

One salient feature of TOM is the fact that despite robust levels of human T cells, they do not develop GVHD. GVHD has been observed in multiple humanized mouse models [19,36]. Some investigators have reported a significance incidence of GVHD leading to death of some of the animals at 25-28 weeks post-humanization [19]. In contrast, we did not notice any of these effects on TOMs at these or subsequent time points (up to 78 weeks longest time point analyzed). The longevity of TOM systemically reconstituted with high levels of human T cells in the absence of GVHD is an important feature of this model.

ART offers significant benefits to HIV infected patients. Our results show that combination ART is able to suppress viral replication in TOM validating this model for the evaluation of the effect of antivirals on HIV replication *in vivo*. As in humans, therapy interruption resulted in rapid viral rebound. Furthermore, we show that human T cells alone are sufficient for the establishment of HIV latency in resting CD4^+^ T cells. Additionally, latently infected cells in TOM can be induced *ex vivo* to produce virus. The frequency of latently infected resting human CD4^+^ T cells in ART suppressed TOM are within the range seen circulating in PB of ART suppressed patients, regardless of when therapy was initiated. T cells represent the major reservoir of latent HIV in humans. Therefore, TOM may represent a unique tool for studies of HIV eradication strategies, as they have latently infected resting CD4^+^ T cells in the complete absence of any myeloid cells.

## Conclusions

In summary, TOM represent a significant advance over the original SCID-hu thy/liv model because they have substantial levels of T cells in both PB and tissues. TOM are systemically and exclusively reconstituted with human T cells enhancing their utility for the study of T cell development, repopulation, function and response to stimuli *in vivo*. The presence of human T cells in blood permit direct inoculations with HIV and direct monitoring of virus infection via blood plasma facilitating the longitudinal analysis of HIV infection and its effects on CD4^+^ T cells. ART efficiently inhibits HIV replication in TOM resulting in strong viral suppression. The ability to suppress HIV replication by ART in TOM allows the use of these mice to investigate latently infected resting human CD4^+^ cells *in vivo*. Because these mice do not develop GVHD and systemic reconstitution with human T cells is sustained at high levels for over a year, long-term experiments are greatly facilitated in this model.

## Methods

### Generation of humanized mice

Humanized TOM were prepared by implanting allogeneic thymus and liver tissue into, 6–8 week old NOD.Cg-Prkdc^scid^ Il2rg^tm1Wjl^/SzJ (NSG, The Jackson Laboratory) mice. NOD/SCID hu-thy/liv (N/S-hu) mice were prepared in the same manner by implanting thymus and liver tissue into NOD.CB17-Prkdc^scid^/J mice (NOD/SCID, The Jackson Laboratory). Seven different tissue sets were used to generate the humanized mice presented in this manuscript. The thymus and liver implants consisted of a 1-2 mm piece of liver tissue sandwiched between two pieces of autologous thymus that were placed under the left kidney capsule (Advanced Bioscience Resources, Alameda, CA). All mice were maintained in a specific pathogen-free facility with the Division of Laboratory Animal Medicine at the University of North Carolina at Chapel Hill (UNC-CH) according to protocols approved by the Institutional Animal Care and Use Committee. Human reconstitution of mice was monitored by flow cytometric analysis for human CD45^+^ cells in peripheral blood, as previously described [37,38]. Peripheral blood samples were obtained via submandibular venipuncture and were collected in tubes containing EDTA. Whole peripheral blood was stained with antibodies, red blood cells were lysed, and the remaining cells were washed and fixed using a 1% paraformaldehyde solution. A total of 10,000-30,000 events were collected per animal at each time point as indicated below.

### Tissue harvesting and flow cytometric analyses of humanized mice

Mononuclear cells (MNCs) were isolated from the bone marrow, spleen, lymph nodes, lung, liver, and thymic organoid as previously described [38]. Tissues were minced and/or digested and filtered through a 70 μm strainer. The liver and lung were processed as previously described [39]. For all latency determinations, mononuclear cells, with the exception of the lymph nodes, were isolated using a Percoll gradient. Red blood cells were lysed as needed (namely for the spleen, bone marrow and liver tissues). MNCs were washed, counted via trypan blue exclusion, and flow cytometric analyses were performed for the indicated markers [13,24,37-39]. Live cells were distinguished by their forward and side scatter profiles as previously described [40]. Flow cytometry data was collected on either a BD FACSCanto or a BD LSRFortessa flow cytometer, and analyzed using BD FACSDiva software (v.5.0.2 or v.6.1.3).

### HIV-1 infections

Stocks of HIV-1_JR-CSF_ were prepared and titered as previously described [41]. Briefly, virus supernatants were prepared via transient transfection of 293 T cells, and were tittered using TZM-bl cells essentially as we have previously described [42]. Parenteral exposures were performed using HIV-1_JR-CSF_ (90,000 TCIU) administered either intravenously or intraperitoneally. A total of two intraperitoneal and six intravenous exposures were performed, yielding 2/2 and 6/6 systemically infected animals, respectively.

### Analysis of HIV-1 infection

Peripheral blood was collected via retro-orbital bleed using EDTA coated capillary tubes (approximately 100 ul total). Infection of TOM with HIV-1 was determined with a one-step reverse transcriptase real-time PCR assay (ABI custom TaqMan Assays-by-design) according to the manufacturer’s instructions (with primers 5′-CATGTTTTCAGCATTATCAGAAGGA-3′ and 5′-TGCTTGATGTCCCCCCACT-3′; assay sensitivity of 400 RNA copies per mL). Additionally, the percent of human CD4^+^ T cells in the peripheral blood of TOM pre- and post-exposure to HIV-1 were monitored by flow cytometry (using 40-60 ul of blood). Changes in the percent of CD4^+^ T cells present in the tissues of infected and uninfected animals were compared by two-way ANOVA, and were not significantly different. Statistical analysis was performed in Prism version 5 (GraphPad Software, Inc., San Diego, CA).

### Antiretroviral treatment of TOM

For HIV treatment we used a previously described triple combination of drugs that we have shown to be effective at suppressing viral load in humanized mice. Specifically, infected TOM were administered daily intraperitoneal injections of emtricitabine (FTC; 140-200 mg/kg), tenofovir disoproxil fumarate (TDF; 146-208 mg/kg) and raltegravir (RAL; 56-80 mg/kg) for six to nine weeks, as previously described [13]. HIV-1 infection was monitored throughout ART as described above.

### Resting cell isolation and latency determinations of TOM and patient samples

All MNCs from individual mice were pooled. Resting human CD4^+^ T cells were isolated from pooled tissues or from leukopheresis product of patient samples using negative magnetic selection (STEMCELL Technologies, Vancouver) as previously described [12,13,43]. Briefly, MNCs obtained from mouse tissues were incubated with a cocktail of antibodies composed of mouse anti CD45 and TER119, and anti-human CD8, CD14, CD16, CD19, CD56, CD41, CD25, CD31, CD105, HLA-DR, and glycophorin A. For the separation of cells from human samples, the mouse antibodies were not included in the isolation cocktail. Antibody-bound cells were removed using a column based-magnetic purification system and the purified resting cells were collected as flow through. This approach resulted in a >99% pure resting CD4^+^ T cell population. Resting CD4^+^ T cells were then cultured with 15 nM efavirenz and 1 μM raltegravir for 2 days prior to performing viral outgrowth assays to prevent any de-novo infection from unintegrated virus [13]. Viral outgrowth was achieved by maximally stimulating resting cells in limiting dilution cultures containing 60 U/ml IL-2, 1ug/ml phytohemagglutinin (PHA) and irradiated allogeneic PBMC from an uninfected donor.al. [12,13]. The culture media was replaced every 3-4 days with fresh media containing 5 U/ml IL-2. CD8 depleted PHA-stimulated PBMC from an uninfected donor were added twice during the experiment to facilitate virus spread/amplification in cultures. Cultures were scored positive if p24 was detectable at day 15 and confirmed on day 19. The number of infected resting cells was estimated by a maximum likelihood method and was expressed as the infectious units per million resting CD4^+^ T cells (IUPM) [12].

## Competing interests

The authors declare that they have no competing interests.

## Authors’ contributions

JBH conceived the study, carried out viral inoculations, tissue and PB collection/processing, flow cytometric analysis and drafted the manuscript. AW also performed viral inoculations, tissue collection/processing, and flow cytometric and immunohistochemical analysis. NA carried out the limiting dilution assay and subsequent IUPM calculations from both patient and mouse samples. SC carried out the magnetic negative selection of resting cells from the pooled tissues of TOM. DM participated in study design and coordination of samples. JVG conceived of the study, participated in the design and coordination, and drafted the manuscript. All authors read and approved the final manuscript.
